# Bacterial microbiome in tropical lichens and the effect of the isolation method on culturable lichen-derived actinobacteria

**DOI:** 10.1038/s41598-023-32759-2

**Published:** 2023-04-04

**Authors:** Trinset Weeraphan, Achiraya Somphong, Vasun Poengsungnoen, Kawinnat Buaruang, Enjuro Harunari, Yasuhiro Igarashi, Somboon Tanasupawat, Wongsakorn Phongsopitanun

**Affiliations:** 1 Department of Biochemistry and Microbiology, Faculty of Pharmaceutical Sciences, Chulalongkorn University, 254 Phayathai Road, Pathumwan, Bangkok, 10330 Thailand; 2 Lichen Research Unit, Department of Biology, Faculty of Science, Ramkhamhaeng University, Bangkok, Thailand; 3 Biotechnology Research Center and Department of Biotechnology, Toyama Prefectural University, Imizu, Toyama Japan; 4 Natural Products and Nanoparticles Research Unit (RP2), Chulalongkorn University, 254 Phayathai Road, Pathumwan, Bangkok, 10330 Thailand

**Keywords:** Ecology, Microbiology, Ecology

## Abstract

Ten samples of tropical lichens collected from Doi Inthanon, Thailand, were explored for the diversity of their bacterial microbiomes through 16S rRNA-based metagenomics analysis. The five predominant lichen-associated bacteria belonged to the phyla *Proteobacteria* (31.84%), *Planctomycetota* (17.08%), *Actinobacteriota* (15.37%), *Verrucomicrobiota* (12.17%), and *Acidobacteriota* (7.87%). The diversity analysis metric showed that *Heterodermia* contained the highest bacterial species richness. Within the lichens, *Ramalina conduplicans* and *Cladonia rappii* showed a distinct bacterial community from the other lichen species. The community of lichen-associated actinobacteria was investigated as a potential source of synthesized biologically active compounds. From the total Operational Taxonomic Units (OTUs) found across the ten different lichen samples, 13.21% were identified as actinobacteria, including the rare actinobacterial genera that are not commonly found, such as *Pseudonocardia*, *Kineosporia*, *Dactylosporangium*, *Amycolatopsis*, *Actinoplanes*, and *Streptosporangium*. Evaluation of the pretreatment method (heat, air-drying, phenol, and flooding) and isolation media used for the culture-dependent actinobacterial isolation revealed that the different pretreatments combined with different isolation media were effective in obtaining several species of actinobacteria. However, metagenomics analyses revealed that there were still several strains, including rare actinobacterial species, that were not isolated. This research strongly suggests that lichens appear to be a promising source for obtaining actinobacteria.

## Introduction

Lichens are symbiotic organisms with a mutualistic relationship between heterotrophic fungi (mycobionts) and photoautotrophic organisms (photobionts), either unicellular green algae or cyanobacteria^1^. This symbiosis occurs when the mycobiont contacts with a suitable photobiont and leads to the developmental process of lichen's unique structure, called the thallus^2^. Although lichens are slow growing organisms (only a few mm per year^3^), they are estimated to cover 8–10% of the planet's land surface^1,4^. Lichens are important to the ecosystem as they are part of soil formation, improve soil quality, and uptake and release minerals and nutrients for plants^5,6^. In addition, lichens are also known as prolific producers of secondary metabolites that could be used as antimicrobial, antioxidant, and cytotoxic substances^7,8^.

Because many lichens can survive in severe nutrient-poor conditions, the lichen-associated bacteria have increasingly been recognized as key participants, where it is assumed that these bacteria could provide some significant source of vital nutrients to lichens^9^. Before the advent of molecular methods, culture-based approaches to the characterization of bacterial communities associated with lichens revealed the probable functional role of nitrogen-fixation as a consequence of various bacteria, such as *Azotobacter*, *Bacillus*, *Clostridium*, and *Pseudomonas*^10^. Recently, next-generation sequencing, including the application of omics technologies, have been employed to reveal the association of diverse bacterial microbiomes in lichens in order to understand their ecological role^11–14^. Despite that, the complexity of these lichen-associated bacteria is not yet well explored and comparing research is complicated by variations in the procedure of sample collection, data analysis, and lichen types or species used^15^.

Actinobacteria are an exceedingly varied group of Gram-positive, filamentous, cytosine- and guanine-rich bacteria^16^. Actinobacteria have been explored as part of the bacteria community within lichens^17^. Several studies have found that actinobacterial species associated with lichens can produce secondary metabolites with medicinal properties, e.g. the discovery of uncialamycin^18^, angucycline, and butenolide from lichen-derived *Streptomyces*^19^. For all the reasons above, coupled with the fact that lichen-associated bacterial communities are still poorly characterized, lichens have become a promising source of microbiota for actinobacterial isolation in the prospect of discovering novel bioactive compounds for pharmaceutical fields^20^.

In this study, the communities of lichen-associated bacteria distributed in 10 different species (eight different genera) of tropical lichens were investigated via metagenomic sequencing through the 16S ribosomal RNA using the Illumina MiSeq platform. The diversity of bacteria, including actinobacteria, was explored and compared using alpha- and beta-diversity analysis based on the appearance of Operational Taxonomic Units (OTUs). A rarefaction curve was used to display the species richness in each sample. The presence of lichen-associated actinobacteria from the microbiome data was compared with the conventional culture-based method to show the limitation of the latter. In addition, since actinobacteria are slow-growing and their spores are more resistant to being incapacitated than most bacteria, we also investigated the effect of various pretreatments (heat, air-drying, phenol, and flooding) to explore the different culturable actinobacteria associated with lichens.

## Results

### Diversity of bacterial community in lichens

**Figure 1:**
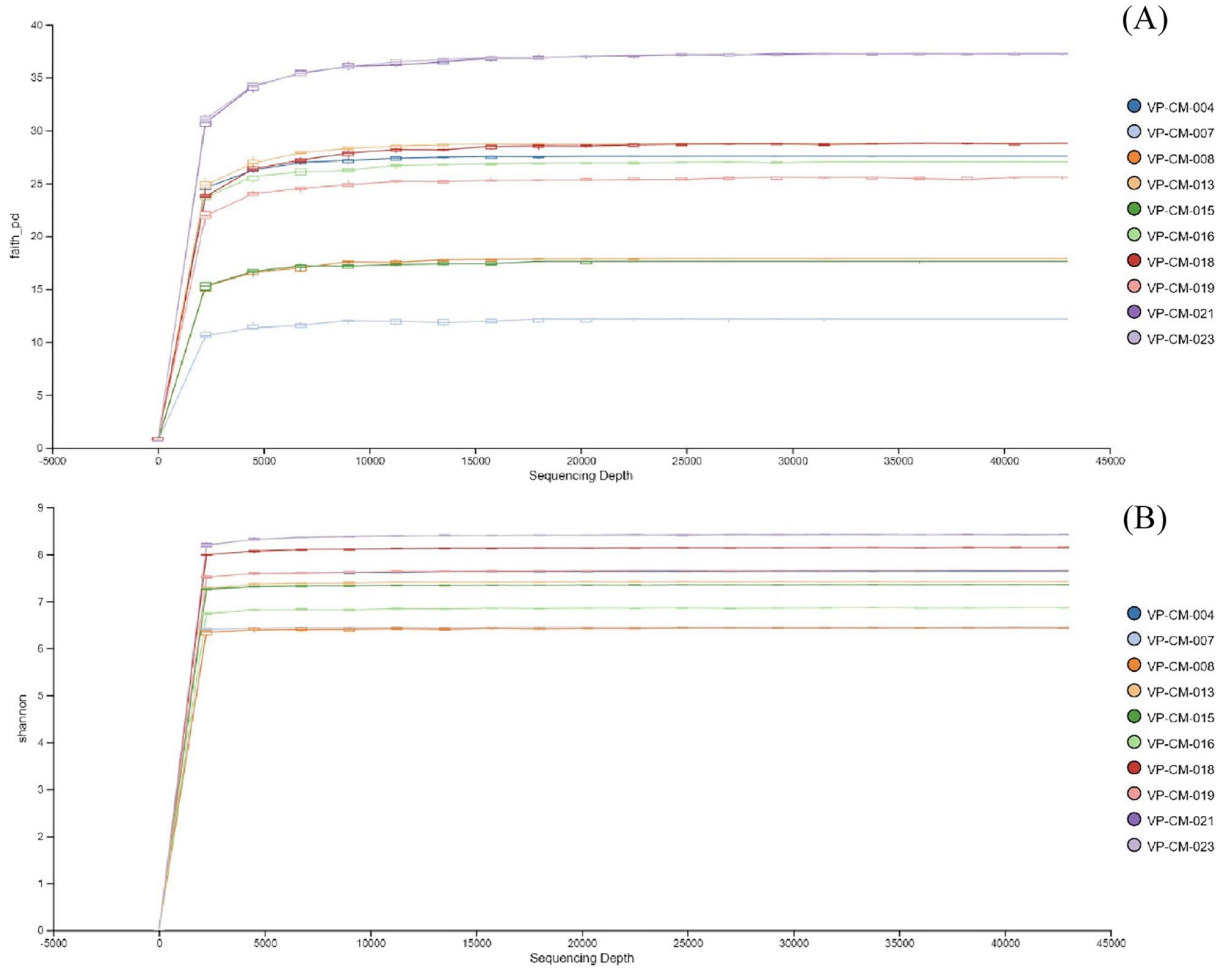
Rarefaction curve showing the bacterial species richness observed from 10 lichen samples, estimated with (**A**) Faith’s PD metric and (**B**) Shannon metric index.

Overall, a total of 427,600 OTUs were observed from all 10 lichen samples, with the principal OTUs in the bacterial microbiomes from all ten lichen species being placed in the phyla *Proteobacteria* (31.84% OTUs), *Planctomycetota* (17.08% OTUs), *Actinobacteriota* (15.37% OTUs), *Verrucomicrobiota* (12.17% OTUs), *Acidobacteriota* (7.87% OTUs), *Chloroflexi* (6.75% OTUs), *Cyanobacteria* (4.66% OTUs), *Bacteroidota* (1.48% OTUs), and *Armatimonadota* (1.08% OTUs) (Supplementary Data 1). At the family level, the top five families found from across all the lichen samples were *Beijerinckiaceae* (18.08% OTUs), *Chthoniobacteraceae* (11.11% OTUs), *Acetobacteraceae* (7.46% OTUs), *Gemmataceae* (6.78% OTUs), and an unidentified family in the order *Tepidisphaerales* (4.97% OTUs) (Supplementary Data 1). When assessing in depth at the genus level, 27 bacterial genera were found across all the samples (Supplementary Data 2). However, many of them were reported as unidentified genera. The richness of bacterial diversity within the lichens was represented as a rarefaction curve and indicated that the sampling was sufficient, as all the samples reached saturation (Fig. 1). The Shannon metric revealed that *Heterodermia diademata* (VP-CM-021) and *Heterodermia lepidota* (VP-CM-023) had the highest species richness. In contrast, samples of *Ramalina conduplicans* (VP-CM-007) and *Parmotrema tinctorum* (VP-CM-008) had the lowest species richness (Fig. 1A). Also, the Faith’s Phylogenetic Diversity metric (Faith’s PD) showed that the bacterial community in the samples of *Heterodermia diademata* (VP-CM-021) and *Heterodermia lepidota* (VP-CM-023) had the highest phylogenetic diversity (Fig. 1B).

**Figure 2:**
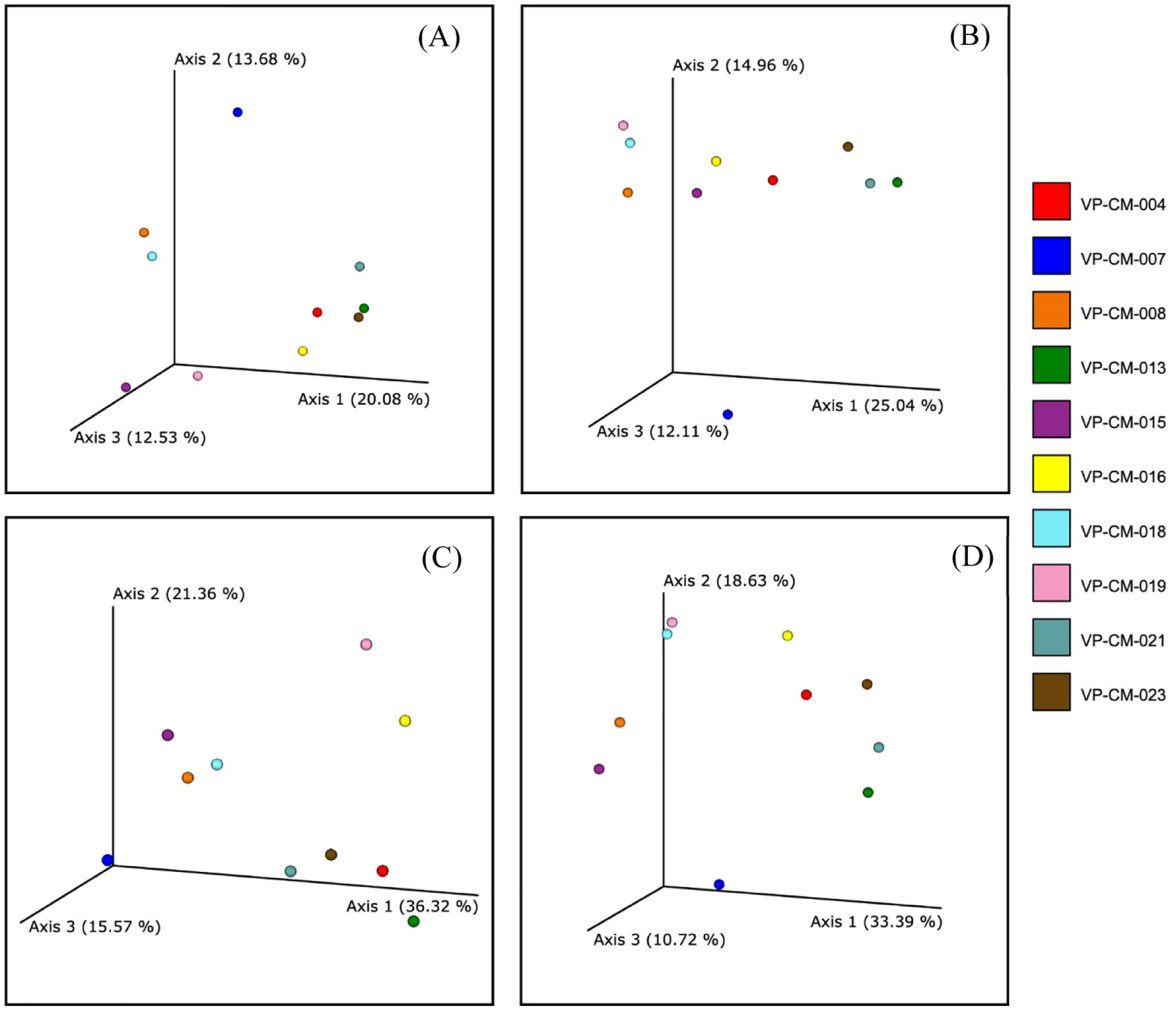
Principal coordinate analysis (PCoA) of the (**A**) Bray–Curtis dissimilarity, (**B**) Jaccard distance matrix, (**C**) weight Unifrac, and (**D**) unweight Unifrac to illustrate the beta-diversity analysis of bacterial communities between ten different lichen species.

**Figure 3:**
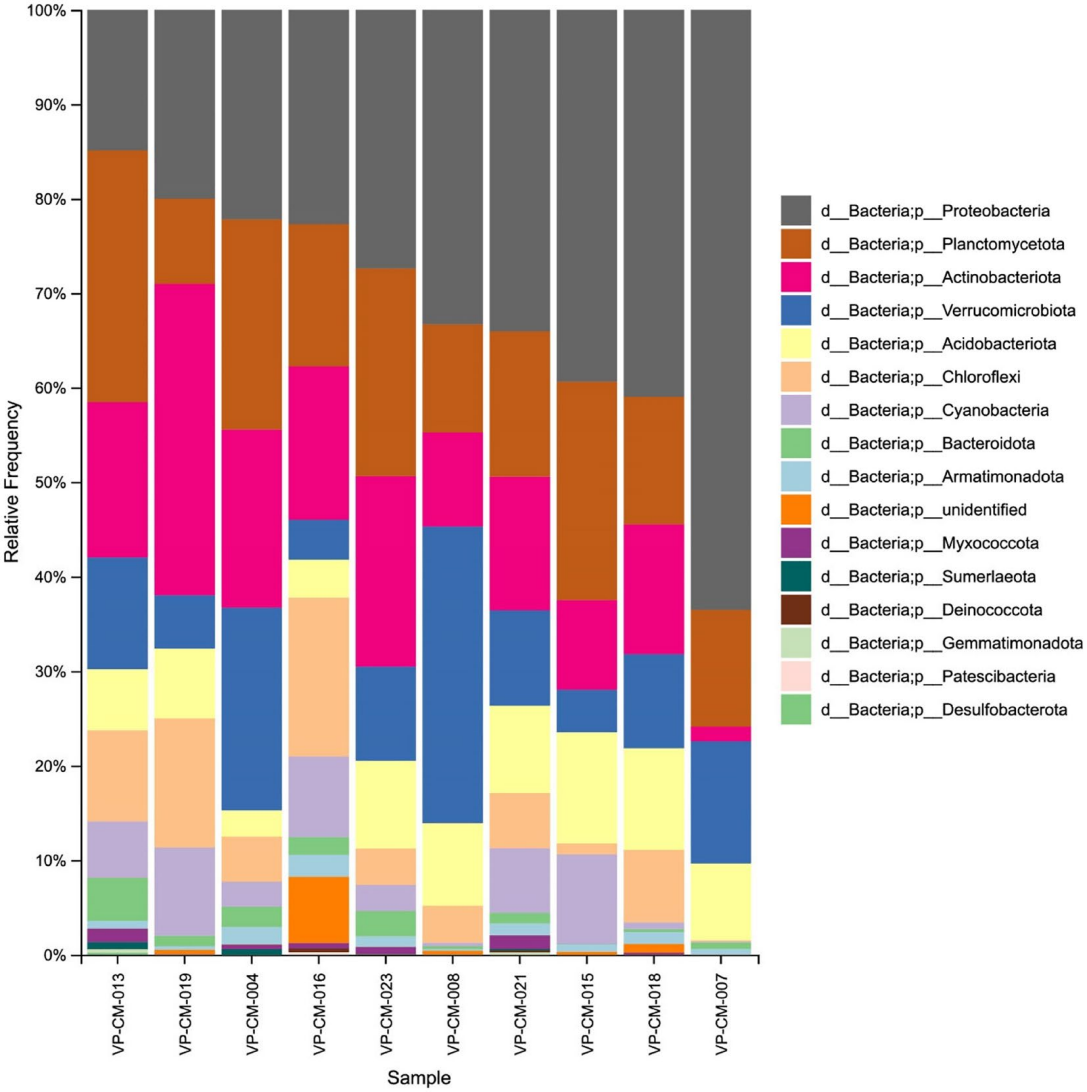
Taxa bar plot representing the relative abundance of bacterial phyla from 10 lichen samples.

As a quantitative and qualitative investigation, the Bray–Curtis dissimilarity and Jaccard distance metrics were employed for the beta-diversity analysis of the lichen-associated bacteria among the different lichen samples. Based on the phylogenetic diversity, both weighted and unweighted UniFrac distances were evaluated. These results indicated that the bacterial communities in samples of *Ramalina conduplicans* (VP-CM-007) and *Cladonia rappii* (VP-CM-015) were distinct from those in the other lichen samples (Fig. 2). The variance of bacteria in each sample was illustrated in a taxa barplot (Fig. 3). Several bacterial genera, for example *Acidothermus*, *Bryobacter*, *Conexibacter*, *Bradyrhizobium*, and *Acidibacter*, were discovered in other lichens but not in *Ramalina conduplicans* (VP-CM-007), which had the lowest diversity richness. In contrast, the bacterial genera *Aridibacter*, *Vicinamibacter*, *Smaragdicoccus*, *Actinoplanes*, *Actinocorallia*, *Lacunisphaera*, and unidentified genus in the family *Blastocatellaceae*, were only found in *Heterodermia diademata* (VP-CM-021) and *Heterodermia lepidota* (VP-CM-023) (Supplementary data 1).

### Analysis of lichen-associated actinobacteria

**Figure 4:**
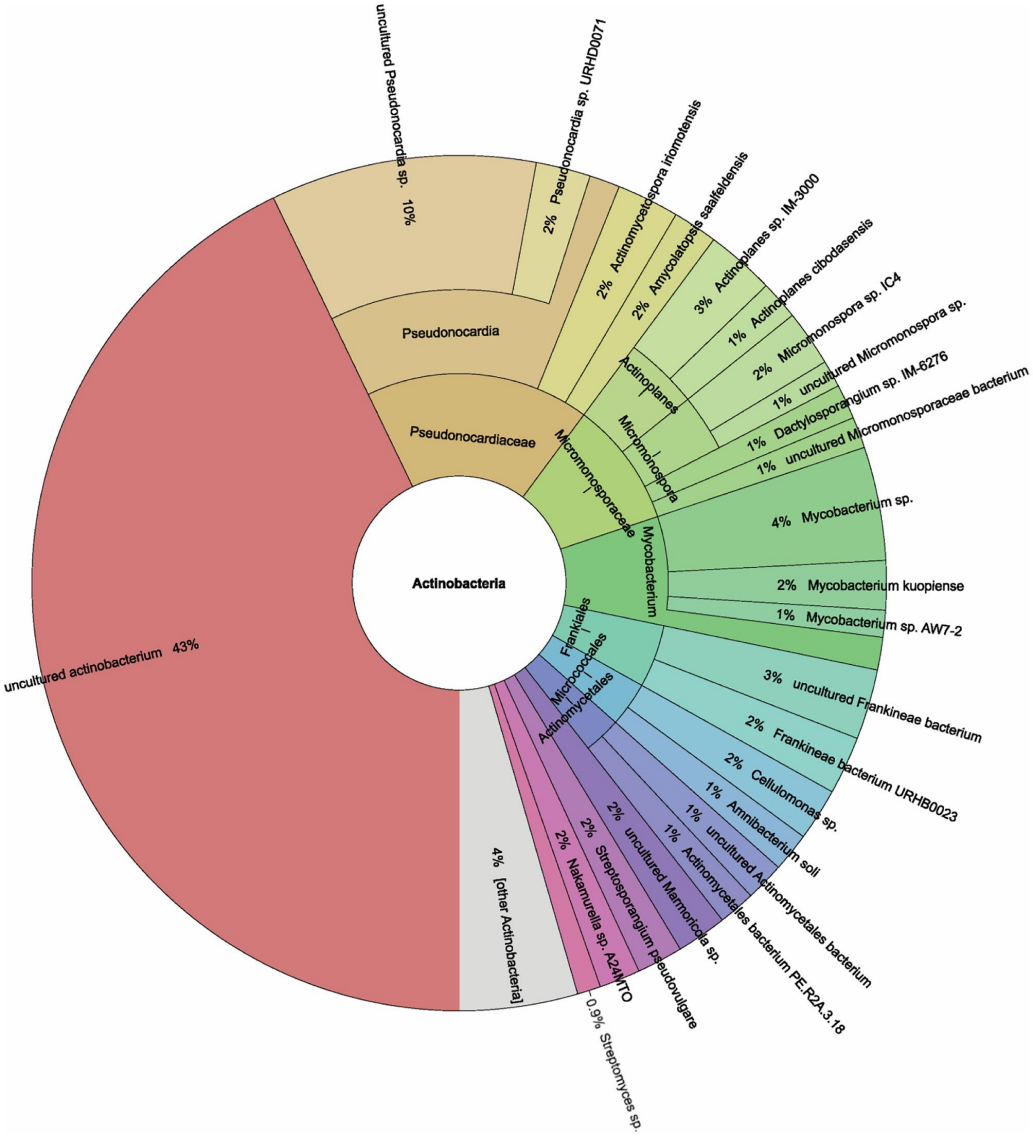
Krona charts visualize the taxonomic spectrum of actinobacteria derived from metagenomic data of the bacterial community found in 10 different lichen species. The circle indicated ascending taxonomic classifications up to the species level (outermost circle).

Analysis of ten lichen species showed that 56,506 OTUs (13.21% of the 427,600 total OTUs) were classified as bacteria in the class *Actinobacteria*, with *Pseudonocardiaceae* having the highest number of OTUs (19,152 or 4.48%). The top five most abundant actinobacteria families, in terms of OTUs, were: *Pseudonocardiaceae*, *Acidothermaceae*, *Micromonosporaceae*, *Mycobacteriaceae*, and *Kineosporiaceae* (Supplementary Data 1). *Parmelinella wallichiana* (VP-CM-019) contained the greatest number of *Actinobacteria* (12,518 OTUs), mostly *Acidothermaceae* (6910 OTUs). In contrast, *Ramalina conduplicans* (VP-CM-007) contained the lowest number of *Actinobacteria* (654 OTUs), which were primarily *Microbacteriaceae* (316 OTUs). At the genus level, the five most frequent genera were *Pseudonocardia* (18,022 OTUs), *Acidothermus* (13,390 OTUs), *Mycobacterium* (5659 OTUs), *Micromonosporaceae* (5103 OTUs), and *Kineosporia* (2212 OTUs) (Supplementary Data 1). For a more comprehensive description of the diversity, Krona Taxonomic Spectrum^21^ was used to illustrate the composition of actinobacteria found in all 10 lichen species at the species level (Fig. 4).

### Isolation of actinobacteria from lichens using culture-dependent method

Based on attempts to isolate actinobacteria from 10 lichen samples using starch-casein agar medium without any pretreatment, we were able to identify 16 actinobacteria isolated from five lichen species (Table 1). The predominant genus was *Streptomyces* (12 isolates), which was found in four lichen species from two genera: *Heterodermia obscurata* (VP-CM-004), *Phaeophyscia hispidula* (VP-CM-013), *Heterodermia diademata* (VP-CM-021), and *Heterodermia lepidota* (VP-CM-023). Nevertheless, some rare actinobacteria were identified, including *Amycolatopsis* and *Streptosporangium* [isolated from *Heterodermia diademata* (VP-CM-021)], while the lichens *Phaeophyscia hispidula* and *Heterodermia diademata* had the highest number of culturable actinobacteria. Although the conventional culturing method without sample pretreatments leads to the success of culture-dependent isolation, the amount of actinobacterial species isolated was still low. Because of this, the effect of the sample pretreatment methods was investigated.

**Table 1:** Identification of the isolated lichen-derived actinobacteria based on 16S rRNA gene sequence.

Identification^a^	Accession number of the closest type strain	Similarity (%)	Isolation sample	Total
*Streptomyces*				
* S. rubidus*	AY876941	98.26	VP-CM-004	1*
* S. seoulensis*	JNXP01000045	99.89 – 100	VP-CM-013	3
* S. olivaceus*	JOFH01000101	98.80	VP-CM-013	1
* S. misionensis*	FNTD01000004	99.79 – 99.80	VP-CM-023	2
* S. paucisporeus*	jgi.1076282	98.59	VP-CM-023	1*
* S. atroolivaceus*	JNXG01000049	99.69	VP-CM-023	1
* S. lutosisoli*	KM000841	99.37	VP-CM-021	1
* S. rhizosphaerihabitans*	HQ267983	99.26	VP-CM-021	1
* S. purpurascens*	AB184859	99	VP-CM-013	1
*Amycolatopsis*				
* A. tolypomycina*	FNSO01000004	98.37	VP-CM-021	1*
* A. saalfeldensis*	DQ792500	97.31	VP-CM-021	1*
*Streptosporangium*				
* S. amethystogenes*	AB537172	98.82	VP-CM-021	1
Total isolated actinobacteria				15

^a^nearest match(es).

*considered as a novel species.

### Effect of sample pretreatment and cultural conditions on the isolation of culturable actinobacteria

**Figure 5:**
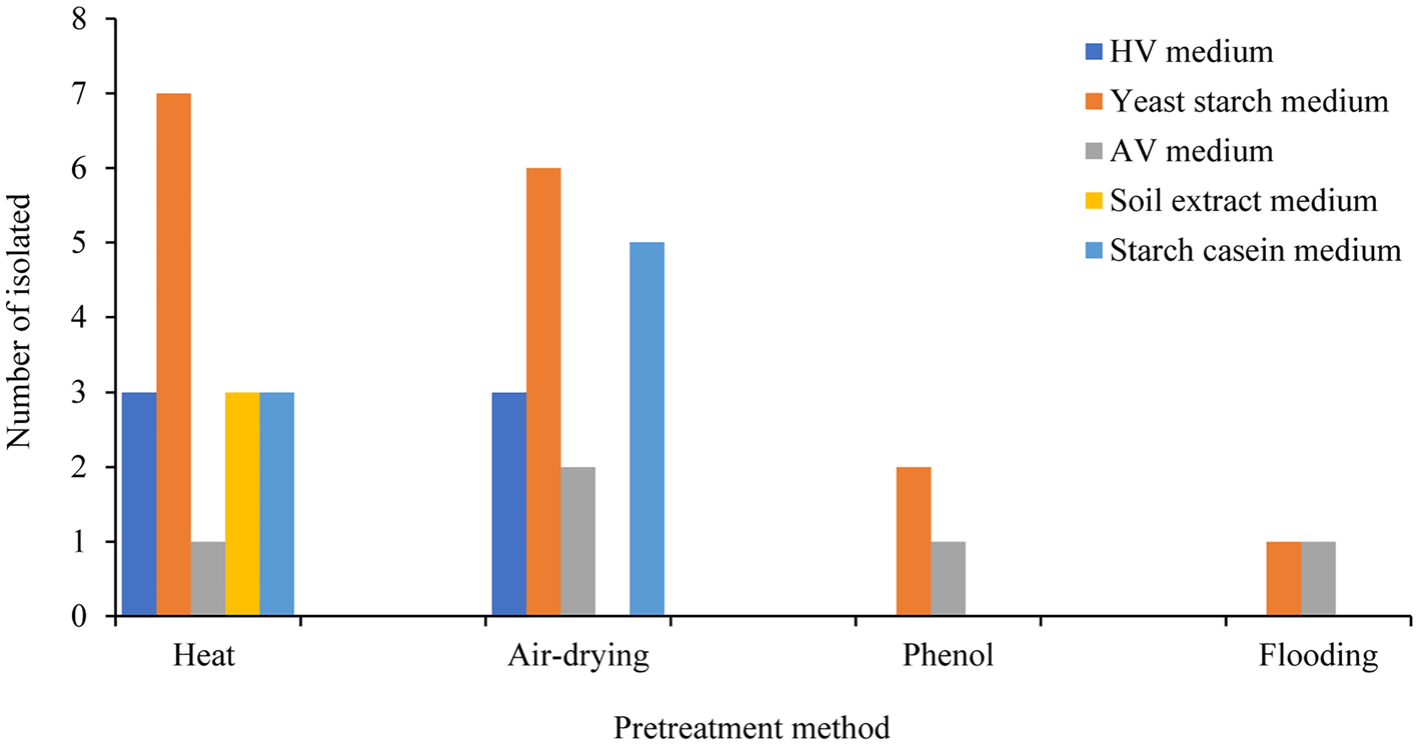
Number of actinobacteria cultured on different isolation media were sorted from differentially pretreated lichens (heat, air-drying, phenol, and flooding).

**Figure 6:**
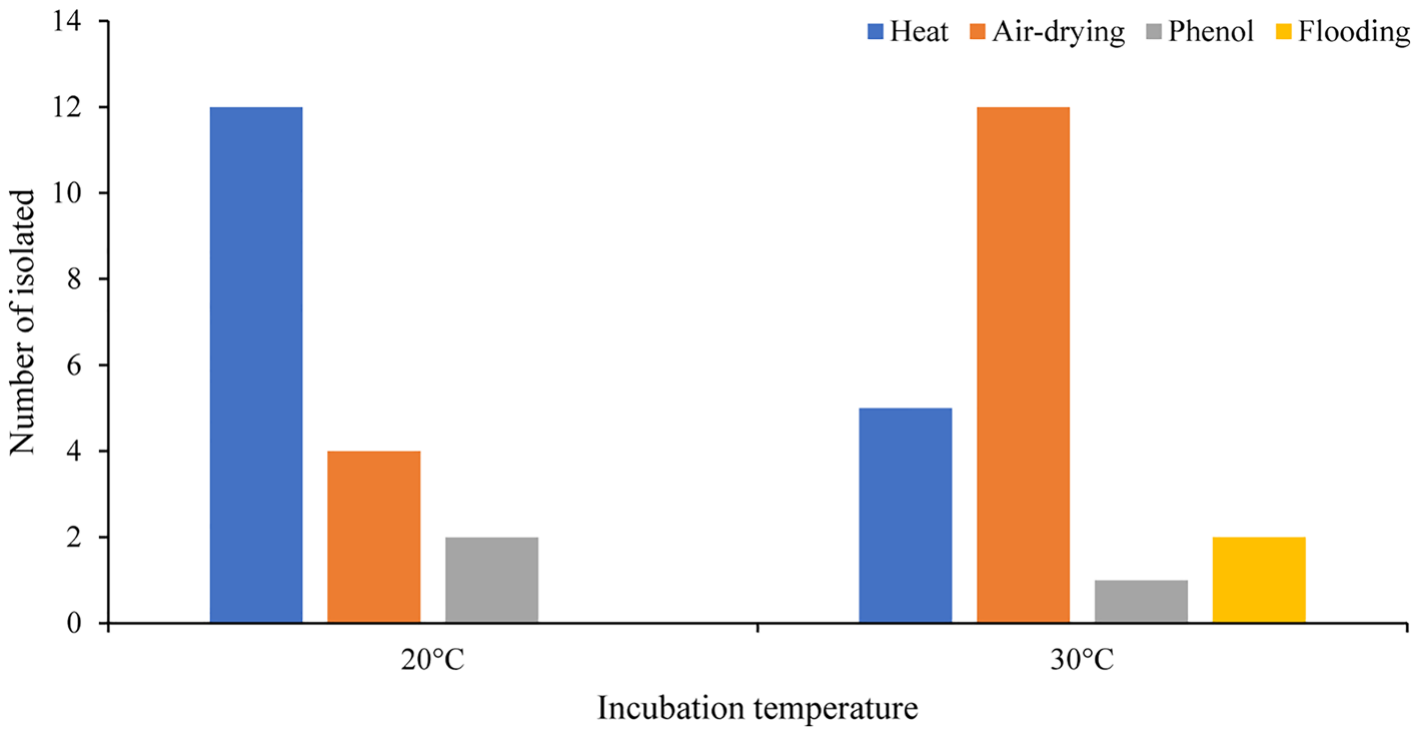
Number of actinobacteria isolated from different incubation temperatures.

To evaluate the effect of pretreatment conditions on the isolation of actinobacteria, the sample of *Heterodermia lepidota* (VP-CM-023) was subjected to several pretreatment techniques. Based on the sequencing of the 16S rRNA gene, a total of 38 actinobacteria were isolated and identified, which belonged to the *Streptomyces* (24 isolates), *Micromonospora* (10 isolates), *Dactylosporangium* (two isolates), *Tsukamurella* (one isolate), and *Streptosporangium* (one isolate) genera (Table 2). The most effective pretreatment method was heat (17 isolates), followed by air-drying (16 isolates), phenol (three isolates), and flooding (two isolates), respectively. The use of different culture media also had an effect on actinobacteria isolation, with yeast starch medium appearing to be the most effective for isolation of actinobacteria from all tested pretreatments (Fig. 5). With respect to the incubation temperature (20 °C and 30 °C), the number of isolated bacteria were broadly the same (18 and 20 isolates, respectively); however, no actinobacteria were obtained at 45 °C of incubation (Fig. 6).

**Table 2:** Identification of the isolated actinobacteria from *Heterodermia lepidota* (VP-CM-023) using different pretreatment methods.

Identification^a^	Accession number of the closest type strain	Similarity (%)	Number of isolates from different pretreatment method				Total
			Heat	Air-drying	Phenol	Flooding	
*Streptomyces*							
* S. rhizosphaerihabitans*	HQ267983	99.15–99.90	6	2	1	0	9
* S. mirabilis*	AB184412	99.62	1	0	0	0	1
* S. misionensis*	FNTD01000004	99.61–99.81	3	7	0	0	10
* S. setonii*	MUNB01000146	99.81	1	0	0	0	1
* S. flavovirens*	AB184834	99.80	1	0	0	0	1
* S. neopeptinius*	EU258679	99.27	1	0	0	0	1
* S. sporoverrucosus*	AB184684	99.89	0	1	0	0	1
*Tsukamurella*							
* T. inchonensis*	X85955	99.82	1	0	0	0	1
*Micromonospora*							
* M. auratinigra*	LT594323	98.50–98.62	2*	0	1*	0	3
* M. fulva*	FJ772077	99.17–99.49	0	2	0	0	2
* M. schwarzwaldensis*	KC517406	99.81–99.90	0	4	0	0	4
* M. humi*	jgi.1058870	99.48	0	0	1	0	1
*Dactylosporangium*							
* D. darangshiense*	FM882231	99.48–99.63	1	0	0	1	2
*Streptosporangium*							
* S. carneum*	X89938	100	0	0	0	1	1
Total isolated actinobacteria							38

^a^nearest match(es).

*considered as novel species.

## Discussion

This comparative study of lichen-associated bacteria found that lichen samples VP-CM-021 and VP-CM-023 had the greatest species richness and phylogenetic diversity. Both belonged to the genus *Heterodermia*, a lichen genus typically found in tropical and subtropical regions^22^. Previous research showed that the most abundant bacteria in the lichen *Heterodermia obscurata* were *Proteobacteria* (> 80%), followed by *Actinobacteria*, *Acidobacteriota*, and *Myxococcota*^23^. Here, we found that the bacterial community in *Heterodermia* was predominated by *Proteobacteria*, but other phyla, such as *Planctomycetota*, *Actinobacteriota*, *Verrucomicrobiota*, and *Acidobacteriota*, also contributed to the majority of the bacterial community in both lichen species within the genus *Heterodermia*. Because of some distinct bacterial properties, such as the psychrotolerance of *Planctomycetes*, which made them commonly found in various low-temperature habitats^24^, differences in bacteria in lichens may be influenced by the age of the thallus, environment, including sampling methodology and sample storage^15,25^.

In addition, *Proteobacteria* were the most abundant phylum across the ten lichen species. In accordance with prior research, the majority of *Proteobacteria* identified in all lichen samples were *Alphaproteobacteria*^17,26–29^. Previous fluorescence in situ hybridization analysis revealed the gathering of *Alphaproteobacteria* on the surface of the lichen thallus, where they were organized as a biofilm-like assemblage^26^. *Alphaproteobacteria* are thought to play an important role in lichen symbiosis, such as nutrient supply, phosphate solubilization, iron mobilization, and nitrogen fixation^28–30^. Although this finding indicated the specificity of *Proteobacteria* in many lichens, the dominant bacteria might be different. *Acidobacteria* were reported to be the dominate bacterial group in siliceous rock-attached lichen samples^9^ and Bacteroidetes in some marine lichens^13^. The differences in bacterial communities among lichens could be attributed to the lichen substrate type, sunlight exposure, and lichen secondary metabolites^31,32^.

Cyanobacteria are another group of microorganisms that have gained attention due to their role in the lichen symbiotic relationship. Our study found that lichen samples *Cladonia rappii* (VP-CM-015), *Parmelinella wallichiana* (VP-CM-019), and *Lobaria retigera* (VP-CM-016) had the highest relative abundance of cyanobacteria (9.48%, 9.35%, and 8.53%, respectively). For the taxa depth, the cyanobacterial family *Nostocaceae* was by far the most prevalent, followed by *Coleofasciculaceae* and *Obscuribacteraceae*. Many members of the *Nostocaceae* family are noted for their ability to engage in diverse symbioses and are commonly observed in cyanolichens^33^. *Lobaria* was classified as a tripartite lichen, where the mycobiont is associated simultaneously with both a green algal and a cyanobacterial photobiont^34^. This symbiotic relationship offers ecological benefits, as the cyanobionts exhibit relatively high rates of nitrogen fixation and the green algal photobiont typically supplies nutrients to the lichen through photosynthesis^35^.

At the lower taxa levels, 27 bacterial genera were revealed as the core microbiome that was found in all the lichen samples. These bacterial genera were identified as members of the *Proteobacteria*, *Verrucomicrobiota*, *Planctomycetota*, *Actinobacteriota*, *Acidobacteriota*, and *Armatimonadota*. Additionally, the beneficial effect of these bacteria for the lichen host has been reported. For example, *Rhizobiales* and *Sphingomonadales* (*Proteobacteria*) encouraged the growth of lichen via the production of auxin and vitamins^36^, *Chthoniobacterales* (*Verrucomicrobiota*) developed as a protector for the lichen microbiome under unfavorable conditions^14^, and *Acidobacteriales* is involved in nutritional supply via turnover of methane to the natural environment^37,38^.

The beta-diversity analysis showed the differences in bacterial communities among these 10 lichen species. The bacterial community in *Ramalina conduplicans* (VP-CM-007) and *Cladonia rappii* (VP-CM-015) were distinct from the other lichens. Interestingly, they were the only fruticose lichens in the study samples. These differences could also be observed in the alpha-diversity analysis. The lichen samples VP-CM-007 and VP-CM-015 showed the lowest species richness within samples, and some bacterial classes, such as *Polyangia*, *Acidimicrobiia*, and *Vampirivibrionia*, which were found in other samples were not found in them. However, there is no relationship between the predominant bacterial strains detected in these two lichens. Prior studies have demonstrated that the bacterial community can differ depending on the growth type of lichens. For example, the composition of bacterial communities from crustose lichens were distinct from those of the foliose and fruticose lichens. While *Alphaproteobacteria* dominated in many foliose lichens, *Acidobacteria* dominated in a crustose lichen, *Ophioparma* sp.^9^. Also, *Hydropunctaria* sp., a crustose lichen that inhabits rocks, was mostly colonized by *Cyanobacteria*, *Actinobacteria*, and *Deinococcus*^39^. This suggests that differences in lichen-associated bacteria may also be attributed to the type of lichen growth.

Analysis of the actinobacterial community in lichens through a metagenomics approach revealed that, overall, 56,505 observed OTUs were assigned to actinobacteria (13.21% of total bacterial OTUs). Many of the actinobacterial OTUs correspond with previous studies^17,40,41^. Several genera of actinobacterial OTUs were found in all the lichen samples studied here. For instance, *Pseudonocardia*, *Mycobacterium*, *Jatrophihabitans*, and *Actinomycetospora*. However, only a few studies have reported the finding of these bacteria from lichens^40,42^. The visualization by Krona at the genus level suggested that a significant portion (over 40%) of the actinobacteria detected in these 10 studied lichen species were believed to be uncultivable strains. However, it is important to note that the classification as uncultivable bacteria may not be definitive, as not all the strains have been attempted to be cultured and the data from environmental studies in genetic databases may not have been thoroughly characterized for each identified bacterial strain. Regardless, this indicated the usefulness of the 16S rRNA metagenomics approach to examine actinobacteria members that were previously not obtainable through culture-dependent methods and so may potentially be uncultivable.

Actinobacteria have gained attention due to their potential to synthesize natural bioactive compounds. Isolation and screening of uncommon strains of actinobacteria can improve the probability of finding novel bioactive compounds. Here, our investigation revealed the presence of previously reported uncommon genera^43^ from the total number of observed actinobacterial OTUs, represented by *Pseudonocardia* (31.89% of total actinobacterial OTUs), *Kineosporia* (3.91% of total actinobacterial OTUs), *Dactylosporangium* (2.60% of total actinobacterial OTUs), *Amycolatopsis* (0.30% of total actinobacterial OTUs), *Actinoplanes*, and *Streptosporangium* (0.17% of total actinobacterial OTUs). This suggests that lichens are likely a promising resource for isolating actinobacteria for biotechnology applications. Until now, the correlation between species of rare actinobacteria and lichens has not been well explored. From our investigation, some rare actinobacteria were discovered in various lichen samples, including *Actinoplanes* from *Heterodermia* (VP-CM-021 and VP-CM-023), and *Streptosporangium* from *Lobaria* (VP-CM-016). Interestingly, the actinobacterial genus *Pseudonocardia* was found to be the most abundant among all the lichen samples in this study. Controlling the variables of the experimental sample and repeated testing may be necessary to establish a relationship between the lichens and actinobacteria.

Isolation of actinobacteria from lichens have been studied for nearly two decades. The preponderance of identified actinobacteria are members of the families *Streptomycetaceae*, *Pseudocardiaceae*, *Micromonosporaceae*, and *Thermomonosporaceae*^17^. Most of the numerous investigations have focused on the cultivation-based method despite the fact that only 1% of the diversity of bacteria can be grown using conventional methods and that the remaining 99% can provide an undiscovered pool of novel antibiotics as well as secondary metabolites has received a lot of attention. It is understood that a culture-dependent approach is biased since bacteria can only be cultivated if their physiological and metabolic requirements can be matched in vitro. This is one of the main drawbacks of culture-dependent methods for measuring bacterial populations^44^. This assertion is supported by the application of metagenomics, which can evaluate the presence of microorganisms via a culture-independent approach^45,46^.

Metagenomics is the study of genomic information derived directly from the environment. This direct sequencing approach has been suggested as the most precise method for determining taxonomic diversity^47^. Metagenomics not only aids in the exploration of biodiversity but also in the discovery of various biologically significant substances by deciphering the metabolic pathways of the species involved in the biosynthesis of novel active compounds, such as antibiotics, non-ribosomal peptide synthetase, and polyketide synthase. This strategy provides the advantages of saving time and lowering the dependency on cultured bacteria in the laboratory, enabling the exploration of bioactive compounds from species that are difficult to cultivate^48^.

This study presented the different strategies for exploring bacterial communities by focusing on actinobacteria isolation in lichens. The lichen *Heterodermia lepidota* (VP-CM-023) was chosen as a source of samples to compare the culture-dependent and culture-independent approaches to studying actinobacteria populations. The OTUs from the metagenomics analysis based on the 16S rRNA gene revealed that *Heterodermia lepidota* (VP-CM-023) was associated with various actinobacteria, including rare actinobacteria from the genera *Micromonospora*, *Pseudonocardia*, *Kineosporia*, *Dactylosporangium*, and *Actinoplanes*. In contrast, using a culture-dependent method without sample pretreatment, only *Streptomyces* was isolated from this sample. When employing the pretreatment process on the same sample, up to six genera, including the rare actinobacteria genera *Micromonospora*, *Dactylosporangium*, and *Streptosporangium*, were isolated. The presence of *Streptosporangium* detected in the cultivation-based method but not as an abundant OTU in the metagenomics analysis raises questions about the possibility of contamination, or differences in the fragments used for the metagenomics and culture analysis. Further investigation with additional samples from this particular specimen and species is necessary to provide a more definitive conclusion. Nonetheless, based on the disparities between the OTUs detected in the metagenomics analysis and isolated strains of actinobacteria, this research demonstrated solid evidence of the limits of the culture-dependent methods for studying bacterial diversity and isolating actinobacteria from natural resources.

From a total of 38 isolated strains, the dominant isolated actinobacteria belong to the genus *Streptomyces* (24 isolates), the largest genus of actinobacteria. *Streptomyces* has received attention and been exploited in the medical field as a consequence of its ability to produce several secondary metabolites with bioactive properties. Several species of *Streptomyces* have been isolated previously from lichens and discovered to produce biomedically natural compounds^18,19^. In this study, *S. misionensis* and *S. rhizosphaerihabitans* were the most common species isolated from *Heterodermia lepidota* (VP-CM-023). Interestingly, *S. rhizosphaerihabitans* was previously discovered in the rhizosphere soil from a bamboo forest^49^, and this is the first study to present the isolation of this species from a lichen sample.

Recent reports have identified unexplored rare actinobacteria as important sources of various bioactive compounds^50^. *Micromonospora*, a prevalent bacterial genus found in various ecosystems, such as soil, fresh water, and muddy soil, was revealed to be the largest group of rare actinobacteria and is significant in clinical research^51,52^. In this study of 10 lichen samples, *Micromonospora* were the second most frequently isolated genus of actinobacteria. Based on 16S rRNA sequencing identification, these isolates belong to the species *M. schwarzwaldensis*, *M. auratinigra*, and *M. fulva*.

Pretreatment is an important step for isolating and screening distinct actinobacteria from environmental samples. In principle, pretreatment strategies focus on the actinobacteria of interest by suppressing or eliminating undesirable microbes. Due to the fact that spores of actinobacteria are resistant to desiccation, drying pretreatments, such as heat or air-drying have been employed. Our results indicated that most actinobacteria were successfully isolated after using drying as the sample pretreatment, whether at a high temperature or at room temperature. This included heat-tolerant spore-forming bacteria, such as *Streptomyces* and *Micromonospora*, in accord with previous research^53,54^. Furthermore, many chemicals can be used to inhibit other microbes and increase the probability to obtain actinobacterial strains^55^. Phenol pretreatment is a physiochemical method for inhibiting microorganisms by rupturing lipid-containing protein membranes, causing cellular leakage and forming hydrogen bonds that disrupt bacterial enzymes. Using 1.5% (v/v) phenol to pretreat soil samples was found to decrease the number of fungi and other bacteria, but increase the number of *Micromonospora*^56,57^. Spore formation is one of the characteristics of various actinobacterial species. Members of rare actinomyces, for example, *Actinoplanes* and *Actinosynnema*, are recognized by motile spores. Consequently, the flooding method was developed and, when using 0.1% skim milk as the flooding solution, improved the motility of motile spores and was useful for isolating rare actinobacteria^58^.

The use of specific isolation media is one approach to achieve actinobacterial isolation. In this study, there was no difference in the specificity of actinobacterial strains that could be isolated with the different types of selection medium utilized. Thus, YS medium successfully isolated most of the actinobacteria from *Heterodermia lepidota* (VP-CM-023). Compared to other culture media for microbial growth, YS medium is simple in nutrients (only yeast extract and soluble starch), but has previously been shown to be a selective medium for isolating actinobacteria from various environmental conditions^59,60^. Although many media have been proposed, some isolation media are nutritionally deficient since actinobacteria have a tendency to survive and proliferate (e.g. tap water or purified agar medium). High C/N ratios are the result of the incorporation of complex carbon and nitrogen sources, such as humic acid, starch, and casein, into the medium^61^. Starch-casein medium is mainly composed of soluble starch and casein as carbon and nitrogen sources, and is used to discover saccharolytic bacteria, including actinobacteria. This medium was previously shown to be able to isolate *Micromonospora*, *Streptomyces*, and *Nocardia* with antimicrobial activity from the soils sampled at different altitudes in Nepal^62^.

In addition to the humic acid vitamin medium, which was designed for the isolation of particularly rare actinomycetes, a medium containing a mixture of vitamins and humic acid suitable for actinobacterial growth has also been developed. Even though the growth rate of actinobacteria is slow, the usual colony morphology was simple due to the color difference between the colonies and the black color of the medium. Humic acid activation of spore germination was thought to be one of the factors that caused an increase in the variety of actinobacterial colonies on the medium^56^. Furthermore, the colonization of actinobacteria on isolation media was prevented by the dominance of other microorganisms. Thus, several researchers have employed antifungal agents and antibiotics in the isolation media to selectively suppress different kinds of organisms. Antifungal drugs have been reported to increase the effectiveness of the medium for bacterial isolation. Since fungi are able to thrive alongside actinomycetes, investigations on actinobacteria have shown fungi-inhibiting antibiotics to be very effective. Similarly, the application of antibiotics could eliminate specific bacteria. For instance, nalidixic acid inhibits the growth of certain Gram-negative and Gram-positive bacteria. Employing several chemical germicides simultaneously can further improve the selectivity for the isolation of actinobacterial species. For example, the combination of nalidixic acid and cycloheximide was found to inhibit the growth of the majority of Gram-negative bacteria and fungi and was suitable for the isolation of numerous actinobacterial species^63^.

## Methods

### Sample collection and preparation

Ten samples of lichen were aseptically collected from Doi Inthanon National Park, Chiang Mai, Thailand. Samples were kept at − 20 °C before use. The collected lichens were identified based on their morphology, anatomy, and secondary metabolites. Thallus morphology was examined using a stereomicroscope (Olympus SZ30). The anatomical characteristics were dissected by hand with a razor blade and examined under a light microscope (Olympus BX41). Secondary lichen substances were characterized by thin-layer chromatography (TLC) with solvent systems A and C^64^. The list of lichen species used in this study is provided in Table 3. In order to maintain the integrity of the lichen microbiome, DNA extraction and 16S rRNA metagenomics sequencing were conducted on the samples without surface sterilization.

**Table 3:** The lichens used in this study.

Number	Sample code	Lichen species
1	VP-CM-004	*Heterodermia obscurata*
2	VP-CM-007	*Ramalina conduplicans*
3	VP-CM-008	*Parmotrema tinctorum*
4	VP-CM-013	*Phaeophyscia hispidula*
5	VP-CM-015	*Cladonia rappii*
6	VP-CM-016	*Lobaria retigera*
7	VP-CM-018	*Hypotrachyna cirrhata*
8	VP-CM-019	*Parmelinella wallichiana*
9	VP-CM-021	*Heterodermia diademata*
10	VP-CM-023	*Heterodermia lepidota*

### DNA extraction and 16S rRNA metagenomics sequencing

DNeasy PowerSoil Pro DNA Kit (Qiagen, USA) was used for extraction of DNA from the lichens. For library preparation, the variable V3-V4 region of the 16S rRNA gene was amplified using 2X sparQ HiFi PCR master Mix (QuantaBio, USA) with 341F (5´—TCGTCGGCAGCGTCAGATGTGTATAAGAGACAGCCTACGGGNGGCWGCAG—3´) and 805R primers (5´— GTCTCGTGGGCTCGGAGATGTGTATAAGAGACAGGACTACHVGGGTATCTAATCC—3´). Thermocycling reactions were performed at 98 °C for 2 min followed by 30 cycles of 98 °C for 20 s, 60 °C for 30 s, and 72 °C for 1 min, and then a final 72 °C for 1 min. After that, the PCR products were purified [sparQ Puremag Beads (QuantaBio, USA)] and subsequently indexed using the Nextera XT index primer (5 μL per 50 μL PCR reaction) for 8–10 cycles of PCR thermal cycling as above. The overhanging adaptors (underlined sequence) were automatically trimmed off. Finally, the PCR products were pooled and diluted to 4 pM of loading concentration. Cluster generation of DNA fragments and paired-end sequencing were performed at the Omics Sciences and Bioinformatics Center (Chulalongkorn University, Bangkok, Thailand) using Illumina MiSeq platform.

### Bioinformatic analysis

The QIIME 2 version 2020.8 software was used to analyze bacterial microbiome informatics^65^. Demultiplexing and quality filtering of raw sequence data were performed using the q2-demux plugin, followed by denoising with DADA2 (through q2-dada2)^66^. The SEPP q2-plugin was used to construct the phylogenetic tree, which placed the short sequences into sepp-refs-gg-13–8.qza as the reference tree^67^. Based on the available OTUs, the within sample (alpha-)diversity was analyzed using Faith’s Phylogenetic Diversity^68^ and Shannon metric^69^. Beta-diversity analysis, including weighted and unweighted UniFrac distance^70,71^, Jaccard distance, Bray‐Curtis dissimilarity, and Principal Coordinate Analysis (PCoA), were analyzed using q2‐diversity. Taxonomy was assigned to amplicon sequence variants (ASVs) by q2‐feature‐classifier with classify-sklearn Naïve Bayes methods against the Silva 13_8 99% OTUs reference sequences^73^.

### Actinobacteria isolation from lichens

Lichen samples were air-dried at room temperature for 7 d. Then, 1 g of dried sample was aseptically ground in a mortar before adding 9 mL of basic-lauryl sulfate solution to make a tenfold dilution. Next, 0.1 mL of the resultant solution was spread on starch-casein agar (10 g/L soluble starch, 0.3 g/L sodium caseinate, 2 g/L KNO_3_, 15 g/L agar, pH 7.0) supplemented with 50 mg/L cycloheximide and 25 mg/L nalidixic acid^73^ and incubated at 30 °C for 14 d. A single colony of actinobacteria was transferred and cloned using the streak plate method on International *Streptomyces* Project-2 medium (ISP2). Each pure culture was preserved on an ISP2 agar slant as working culture.

### Effect of pretreatment on actinobacterial isolation

The effect of various pretreatment methods on actinobacterial isolation were investigated using the lichen *Heterodermia lepidota* (VP-CM-023). The sample (0.25 g) was thoroughly ground with a mortar prior to various pretreatment methods (heat, air-drying, phenol, and flooding method), as listed in Table 4. After that, the pretreated sample was suspended in basic lauryl sulfate for serial dilution, and subsequently spread on humic acid vitamin agar (HV), starch-casein agar, yeast starch agar, soil extract agar, and arginine-vitamin agar (AV), containing 25 mg/L nalidixic acid and 50 mg/L cycloheximide, and incubated at 20, 30, and 45 °C for 14 d. Colonies expected to be actinobacteria were selected and cloned using the streak plate technique on ISP2 medium. After incubation at 30 °C for 2 weeks, the purified colonies were preserved on an ISP2 agar slant for short-term storage, and as lyophilized cells in 10% (w/v) skim milk for long-term storage.

**Table 4:** Pretreatment conditions.

Method	Pretreatment condition
Heat	Incubation in an oven at 110 °C for 60 min
Air-drying	Air-dried at room temperature for 60 min
Phenol	Suspended in 1 mL of 1.5% (v/v) phenol at room temperature for 30 min
Flooding	Suspended in1 mL of 0.1% (w/v) skim milk at room temperature for 30 min

### Identification of isolated actinobacteria

The isolates were cultured in ISP2 broth at 30 °C for 5 d and then DNA extraction was performed using a PureLink™ Genomic DNA Mini Kit (Thermo Fisher Scientific, USA). Amplification of the 16S rRNA gene sequence was performed by PCR using the 20F (5′-GAGTTTGATCCTGGCTCAG-3′) and 1500R (5′-GTTACCTTGTTACGACTT-3′) primers^74^. The PCR thermal cycling was performed at 94 °C for 3 min, followed by 30 cycles at 94 °C for 1 min, 50 °C for 2 min, and 72 °C for 2 min, and then followed by a final 72 °C for 7 min. The PCR product was purified by GenepHlow™ PCR Cleanup Kit (Geneaid, Taiwan) before being commercially sequenced using universal primers (Macrogen, Korea). The obtained 16S rRNA gene sequences were aligned and analyzed using the BioEdit Sequence Alignment Editor software then compared to DNA sequence available in NCBI GenBank database using the Basic Local Alignment Search Tool (BLAST) analysis.

## Supplementary Information

Supplementary Information 1.

Supplementary Information 2.

Supplementary Information 3.

## Data Availability

The authors confirm that the DNA sequence data in this study have been deposited in the National Center for Biotechnology Information (NCBI) database (https://www.ncbi.nlm.nih.gov/). The accession numbers and direct links of the bacterial DNA sequences are provided in Supplementary data 3. Raw data that support the findings of this study are available from authors.
