# Supplementary material for: Bacterial microbiome in tropical lichens and the effect of the isolation method on culturable lichen-derived actinobacteria
Source: Sci Rep. 2023 Apr 4;13:5483. doi: 10.1038/s41598-023-32759-2 (PMC10073151; doi:10.1038/s41598-023-32759-2)
Supplement: Supplementary file 2 — Supplementary Information 2. [file 41598_2023_32759_MOESM2_ESM.docx]

Supplementary data 2: List of bacterial genus OTUs observed across all samples

| Phylum | Class | Order | Family | Genus | OTUs observed |
| --- | --- | --- | --- | --- | --- |
| *Proteobacteria* | *Alphaproteobacteria* | *Rhizobiales* | *Beijerinckiaceae* | unidentified | 40968 |
|  |  |  |  | *Methylorubrum* | 14336 |
|  |  |  |  | unidentified | 12060 |
|  |  |  |  | *Methylocella* | 8583 |
|  |  |  |  | uncultured | 508 |
|  |  |  |  |  |  |
|  |  | *Acetobacterales* | *Acetobacteraceae* | unidentified | 19792 |
|  |  |  |  | *Acidiphilium* | 7739 |
|  |  |  |  | uncultured | 2052 |
|  |  |  |  |  |  |
|  |  | *Sphingomonadales* | *Sphingomonadaceae* | *Sphingomonas* | 5930 |
|  |  |  |  | unidentified | 2659 |
|  |  |  |  |  |  |
|  |  | *Elsterales* | uncultured | uncultured | 937 |
|  |  |  |  |  |  |

Supplementary data 2 (cont.): List of bacterial genus OTUs observed across all samples

| Phylum | Class | Order | Family | Genus | OTUs observed |
| --- | --- | --- | --- | --- | --- |
| *Verrucomicrobiota* | *Verrucomicrobiae* | *Chthoniobacterales* | *Chthoniobacteraceae* | unidentified | 36135 |
|  |  |  |  | unidentified | 4127 |
|  |  |  | unidentified | unidentified | 1967 |
| *Actinobacteriota* | *Actinobacteria* | *Pseudonocardiales* | *Pseudonocardiaceae* | *Pseudonocardia* | 18022 |
|  |  |  |  | *Actinomycetospora* | 958 |
|  |  | *Corynebacteriales* | *Mycobacteriaceae* | *Mycobacterium* | 5659 |
|  |  | *Frankiales* | *Frankiaceae* | *Jatrophihabitans* | 2044 |
|  |  |  |  |  |  |
|  | *Thermoleophilia* | *Solirubrobacterales* | unidentified | unidentified | 2650 |

Supplementary data 2 (cont.): List of bacterial genus OTUs observed across all samples

| Phylum | Class | Order | Family | Genus | OTUs observed |
| --- | --- | --- | --- | --- | --- |
| *Planctomycetota* | *Phycisphaerae* | *Tepidisphaerales* | unidentified | unidentified | 21235 |
|  |  |  | *Tepidisphaeraceae* | *Tepidisphaera* | 3430 |
|  |  |  |  |  |  |
|  | *Planctomycetes* | *Gemmatales* | *Gemmataceae* | *Gemmata* | 17339 |
|  |  |  |  | uncultured | 4287 |
|  |  | *Isosphaerales* | *Isosphaeraceae* | *Tundrisphaera* | 9770 |
| *Acidobacteriota* | *Acidobacteriae* | *Acidobacteriales* | *Acidobacteriaceae* | *Terriglobus* | 4918 |
| *Armatimonadota* | *Armatimonadia* | *Armatimonadales* | *Armatimonadaceae* | *Armatimonas* | 1964 |
|  | *Chthonomonadetes* | *Chthonomonadales* | *Chthonomonadaceae* | *Chthonomonas* | 1917 |
